# Reporting of noninferiority and equivalence randomized trials for major prostaglandins: A systematic survey of the ophthalmology literature

**DOI:** 10.1186/1745-6215-9-69

**Published:** 2008-12-03

**Authors:** Oghenowede Eyawo, Chia-Wen Lee, Beth Rachlis, Edward J Mills

**Affiliations:** 1 Faculty of Health Sciences, Simon Fraser University, Burnaby, Canada; 2 Department of Outcomes Research and Evidence Based Medicine, Pfizer Ltd., Surrey, UK; 3 Department of Public Health, University of Toronto, Toronto, Canada; 4 Department of Clinical Epidemiology & Biostatistics, McMaster University, Hamilton, Canada

## Abstract

**Background:**

Standards for reporting clinical trials have improved the transparency of patient-important research. The Consolidated Standards of Reporting Trials (CONSORT) published an extension to address noninferiority and equivalence trials. We aimed to determine the reporting quality of prostaglandin noninferiority and equivalence trials in the treatment of glaucoma.

**Methods:**

We searched, independently and in duplicate, 6 electronic databases for eligible trials evaluating prostaglandins. We abstracted data on reporting of methodological criteria, including reporting of per-protocol [PP] and intention-to-treat [ITT] analysis, sample size estimation with margins, type of statistical analysis conducted, efficacy summaries, and use of hyperemia measures.

**Results:**

Trials involving the four major prostaglandin groups (latanoprost, travoprost, bimatoprost, unoprostone) were analyzed. We included 36 noninferiority and 11 equivalence trials. Seventeen out of the included 47 trials (36%, 95% Confidence Intervals [CI]: 24–51) were crossover designs. Only 3 studies (6%, 95% CI: 2–17) reported a presented results of both ITT and PP populations. Twelve studies (26%, 95% CI: 15–39) presented only ITT results but mentioned that PP population had similar results. Thirteen trials (28%, 95% CI: 17–42) presented only PP results with no mention of ITT population results while 17 studies (36%, 95% CI: 24–51) presented only ITT results with no mention of PP population results. Thirty-four (72%, 95% CI: 58–83) of studies adequately described their margin of noninferiority/equivalence. Sequence generation was reported in 22/47 trials (47%, 95% CI: 33–61). Allocation concealment was reported in only 10/47 (21%, 95% CI: 12–35) of the trials. Thirty-five studies (74%, 95% CI: 60–85) employed masking of at least two groups, 4/47 (9%, 95% CI: 3–20) masked only patients and 8/47 (17%, 95% CI: 9–30) were open label studies. Eight (17%, 95% CI: 9–30) of the 47 trials employed a combined test of noninferiority and superiority. We also found 6 differing methods of evaluating hyperemia.

**Conclusion:**

The quality of reporting noninferiority/equivalency trials in the field of glaucoma is markedly heterogeneous. The adoption of the extended CONSORT statement by journals will potentially improve the transparency of this field.

## Background

There is emerging debate about the adherence to standards or recommendations made by regulatory bodies in the design, statistical analysis, description, and reporting of randomised clinical trials (RCT) intended to show noninferiority or equivalence [1]. The recent extension of the Consolidated Standards of Reporting Trials (CONSORT) statement to noninferiority and equivalence trials [1], the U. S. Food and Drug Administration (FDA) guidelines [2], as well as statements from other regulatory authorities, have updated recommendations to guide the conduct and reporting of RCTs investigating noninferiority or equivalence [3].

Noninferiority and equivalence studies aim to demonstrate that a new experimental treatment is not statistically worse than the active control intervention by more than a pre-specified amount (Δ), the noninferiority or equivalence margin. There are now a large number of studies intending to demonstrate noninferiority or equivalence. However, only very few of them have been designed, described, and reported as such [4]. In designs intending to show noninferiority (i.e. that a new treatment is at least as good as or no worse than an existing treatment) or equivalence (i.e. show that a new treatment has equal or comparable efficacy effects), one area of debate has been the application of the intent-to-treat (ITT) principle versus the per-protocol (PP) analysis [5]. There has been an expectation that ITT analyses are less conservative, tending to reduce the observed treatment difference in noninferiority trials, thus permitting wider confidence intervals; a factor that has increased the dependence on per-protocol analysis. The International Conference on Harmonization of Technical Requirements for Registration of Pharmaceuticals for Human Use (ICH) E9 guideline on 'statistical principles for clinical trials' unequivocally states that the use of the ITT analysis in equivalence and noninferiority trials is 'generally not conservative and its role should be considered very carefully' [6]. However, the Committee for Proprietary Medicinal Products (CPMP) consider both analyses to be equally important [7] and simulation studies have not demonstrated the anticonservative approach of ITT versus PP [8]. In the extension of the CONSORT statement, conducting and reporting PP analyses alongside ITT has been suggested as a means of increasing confidence in the study finding particularly when both methods produce similar results.

In order to determine the methodological quality and reporting standards for those trials designed to show noninferiority or therapeutic equivalence, we conducted a systematic survey of the literature to identify noninferiority and equivalence randomised clinical trials involving glaucoma drugs of prostaglandin origin. We applied the extended CONSORT statement for non-inferiority and equivalence trials as methodological criteria of minimum reporting standards [1].

## Methods

### Eligibility Criteria

Noninferiority and equivalence randomized clinical trials that involved the application of any of the four major drugs of the prostaglandin analogues (latanoprost, travoprost, bimatoprost, and unoprostone) in the treatment of patients with open angle glaucoma (POAG) or ocular hypertension (OH) were eligible for the study. We defined noninferiority trials as 1-sided trials aiming to demonstrate effectiveness not lower than Δ. Whereas, equivalence studies were defined as 2-sided trials wherein the difference between treatments is between -Δ and +Δ margins of equivalence. We further determined a trial to be eligible for inclusion if 'equivalence' or 'noninferiority' was mentioned as the intent anywhere in the methods sections of the manuscripts. We excluded bioequivalence (pharmacokinetic and pharmacodynamic) trials and trials designed to show superiority.

### Search Strategy

Using independent and duplicate searchers (OE, EM), we searched the following 6 databases from inception to March 2008: MEDLINE via PubMed, CinAhl, AMED, Toxnet, Cochrane CENTRAL and E-Psyche. Our MEDLINE search strategy identified the exact MeSH terms for the following expressions: open angle glaucoma, ocular hypertension and prostaglandin* (latanoprost, bimatoprost, travoprost aand unoprostone). We conducted 3 main searches. First we collated all articles with the MeSH key terms "glaucoma, open angle OR ocular hypertension" [see Additional file 1]. We then extracted articles containing the MeSH terms "prostaglandin* OR latanoprost OR travoprost OR bimatoprost OR unoprostone." We then pooled these two searches to produce a third list, representing all articles that had as its key terms, "POAG or OH or prostaglandin* OR latanoprost OR travoprost OR bimatoprost OR unoprostone." We also searched multiple databases including CinAhl, AMED, Toxnet, Cochrane CENTRAL and E-Psyche using key search terms like latanoprost, bimatoprost, travoprost, unoprostone, open angle glaucoma, ocular hypertension and prostaglandin*. Simultaneous review of these databases was possible through the use of the Ovid interdisciplinary database that permits the checking of multiple databases. Where full text was unavailable, we searched Google Scholar and also used interlibrary loans to order full text articles. We supplemented this search by reviewing the bibliographies of key papers.

### Study selection

Working independently and in duplicate OE and EM reviewed all abstracts and full articles, where available, of our search results. We excluded articles at this stage that were not randomized trials or were reviews and/or comments. All full text articles of identified RCT studies that examined treatment with prostaglandin drugs were sought. Eligibility was determined in consensus. Only comparisons containing at least one study drug of interest (i.e., bimatoprost, latanoprost, travoprost or unoprostone) were eligible for inclusion. When comparisons involved several different dosages (e.g., 0.005% or 0.03%), we included only those that met FDA-approved standards. Where we were unsure of specific intentions of noninferiority/equivalence compared to superiority, we contacted one author via email.

### Data Collection

We collected information about the type of interventions tested, study design and purpose. We also obtained data on sample size estimation with margins, type of statistical analysis conducted, population analyzed [ITT or/and PP], efficacy summaries, and use of hyperemia measures. Our study evaluation included an assessment of the following general methodological quality features: allocation concealment, sequence generation, masking status, and the handling of losses to follow-up. We also recorded sample size (recruited, randomized and completed) and the use of a combined test of noninferiority and superiority. Finally, we noted whether there was a clear objective/hypothesis relating to noninferiority or equivalence, a tabular presentation of baseline data (demographic and clinical), and a predetermined noninferiority/equivalence margin.

### Data Analysis

In order to assess inter-rater reliability on inclusion of articles, we calculated the *Phi *statistic, which provides a measure of inter-observer agreement independent of chance [9]. In order to determine the proportion reporting the specific item with appropriate confidence intervals, we first stabilized the variances of the raw proportions (r/n) using a Freeman-Tukey type arcsine square root transformation: y = arcsine (square root(r/(n+1))+arcsine(square root(r+1)/(n+1))), with a variance of 1/(n+1), where n is the denominator overall size [10]. We used StatsDirect (version 2.5.2,  for all calculations.

## Results

### Results of literature search

**Figure 1:**
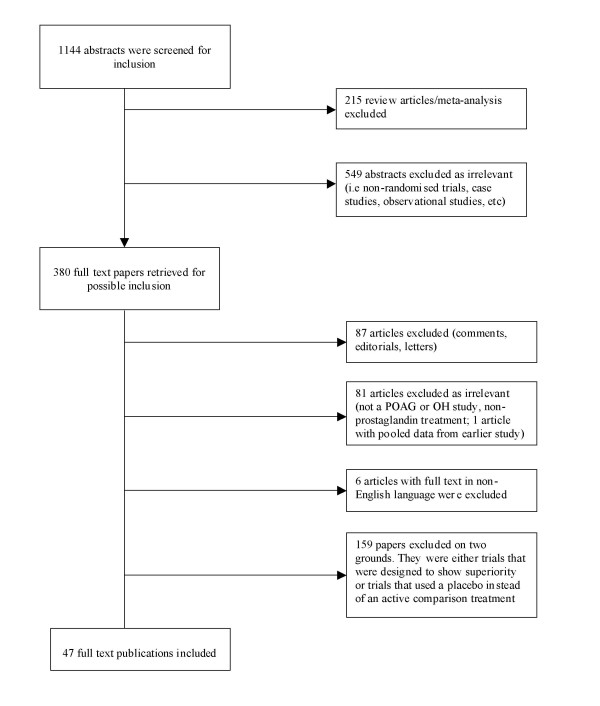
Flow diagram of included studies

Our first and second search produced 35,207 and 97,523 abstracts respectively. The third and final search (search #1 AND #2 pooled) produced 1144 abstracts. After thorough assessment, 215 abstracts were excluded since they were review articles. Another 549 abstracts were excluded as they were not relevant to present study. Overall, 380 full text papers were retrieved for possible inclusion. Upon careful review of the 380 full text articles, we included 47 full text articles in our analysis (Phi = 0.88). Figure 1 presents details of the exclusion criteria at the various stages during the study selection process.

### Trial interventions

Table 1 outlines the characteristics of all 47 included publications. Of these, 31 reported conducting comparisons of treatments (e.g., travoprost vs. latanoprost) that were duplicated in at least one other included study. More specifically, five trials compared bimatoprost to latanoprost [11-15]. Four trials compared bimatoprost and timolol [16-19]. Three trials compared bimatoprost to a fixed combination (FC) treatment that contained timolol [20-22].

**Table 1:** Characteristics of included publications

**Author**	**Year**	**Sample Size** **(N completed)**	**Treatment Comparison**
Topouzis *et al *[40]	2007	332	Travoprost 0.004%/timolol 0.5% versus once-daily latanoprost 0.005%/timolol 0.5%
Hommer [43]	2007	430	FC bimatoprost 0.03%/timolol 0.5% versus non-FC bimatoprost 0.03%/timolol 0.5%
Hollo *et al *[44]	2006	192	Timolol maleate versus brinzolamide *both added to travoprost*
Brittain *et al *[20]	2006	46	Combination timolol 0.5% and latanoprost 0.005% versus bimatoprost 0.03%
Day *et al *[21]	2005	32	bimatoprost 0.03% versus timolol maleate 0.5%/dorzolamide 2% FC
Netland *et al *[45]	2003	25	Brimonidine purite + bimatoprost versus timolol + latanoprost
Thomas *et al *[28]	2003	26	Latanoprost versus brimonidine
Whitcup *et al *[16]	2003	550	Bimatoprost versus timolol
Netland *et al *[23]	2001	760	Travoprost versus latanoprost versus timolol
Larsson [24]	2001	24	Latanoprost 0.005% versus timolol gel-forming solution 0.5%
Fechtner *et al *[42]	2004	238	FC dorzolamide 2%/timolol 0.5% (COSOPT™) versus latanoprost 0.005% (XALATAN™)
Cohen *et al *[17]	2004	284	Bimatoprost versus timolol
Shin *et al *[31]	2004	242	FC latanoprost/timolol versus FC dorzolamide/timolol
Tomita *et al *[25]	2004	39	Latanoprost versus timolol
Stewart *et al *[46]	2004	32	Latanoprost/timolol maleate FC versus concomitant brimonidine and latanoprost therapy
Gandolfi *et al *[11]	2001	214	Bimatoprost versus latanoprost
Watson *et al *[26]	1996	268	Latanoprost versus timolol
Konstas *et al *[57]	2002	35	Once-daily morning versus evening dosing of concomitant latanoprost/timolol
Day *et al *[47]	2003	31	Timolol 0.5%/Dorzolamide 2% FC versus timolol maleate 0.5% and unoprostone...
Parrish *et al *[12]	2003	410	Latanoprost versus bimatoprost versus Travoprost
Konstas *et al *[32]	2004	32	FC latanoprost 0.005%/timolol maleate 0.5% versus FC dorzolamide 2%/timolol maleate 0.5%
Sharpe *et al *[48]	2005	29	Brimonidine 0.2% versus unoprostone 0.15% *both added to timolol maleate 0.5%*
Higginbotham *et al *[18]	2002	1009	Bimatoprost versus timolol
Pfeiffer [33]	2002	396	FC latanoprost and timolol versus its individual components
Higginbotham *et al *[34]	2002	345	FC latanoprost and timolol versus monotherapy with either components
Konstas *et al *[49]	2008	53	Latanoprost and dorzolamide/timolol FC after 2 and 6 months of treatment
Konstas *et al *[37]	2007	40	Latanoprost versus travoprost
Franks *et al *[39]	2006	106	Travoprost 0.004% versus FC latanoprost 0.005%/timolol 0.5%
Diestelhorst *et al *[35]	2006	502	FC latanoprost and timolol in the evening versus concomitant use of the individual components
Hughes *et al *[41]	2005	293	FC travoprost 0.004%/timolol 0.5% ophthalmic solution versus concomitant use of the individual components
Schuman *et al *[50]	2005	387	FC travoprost 0.004%/timolol 0.5% versus concomitant travoprost 0.004% + timolol 0.5 versus timolol BID control
Konstas *et al *[29]	2005	31	Brimonidine purite versus dorzolamide *each added to latanoprost*
Diestelhorst *et al *[36]	2004	190	FC latanoprost/timolol versus concomitant latanoprost and timolol
Coleman *et al *[22]	2003	168	Bimatoprost (LUMIGAN) versus combined timolol and dorzolamide (COSOPT)
Zimmerman *et al *[13]	2003	2775	Various POAG monotherapy versus latanoprost
Stewart *et al *[51]	2003	29	FC timolol maleate/latanoprost versus timolol maleate + brimonidine
Noecker *et al *[14]	2003	249	Bimatoprost versus latanoprost
Honrubia *et al *[52]	2002	217	Latanoprost 0.005% versus FC dorzolamide 2% & timolol 0.5%
Simmons *et al *[30]	2002	107	Brimonidine versus latanoprost in patients uncontrolled on β – blockers
Goldberg *et al *[38]	2001	507	Topical travoprost versus timolol BID
DuBiner *et al *[15]	2001	59	Bimatoprost versus latanoprost
Brandt *et al *[19]	2001	556	QD, BID bimatoprost versus timolol BID
Susanna *et al *[53]	2001	98	Latanoprost versus unoprostone
Diestelhorst *et al *[54]	2000	179	Latanoprost versus pilocarpine T.I.D
Stewart *et al *[55]	1999	30	Timolol solution versus timolol gel *each added to latanoprost*
Konstas *et al *[27]	1999	34	Latanoprost versus timolol maleate
Petounis *et al *[56]	2001	148	Latanoprost versus dorzolamide each added to timolol

Six trials compared latanoprost to timolol [13,23-27]. Three studies compared latanoprost to brimonidine (or brimonidine-containing fixed combination) [28-30]. Two studies compared FC latanoprost/timolol vs FC dorzolamide/timolol [31,32]. Two studies compared FC latanoprost/timolol versus its individual components (i.e. latanoprost and timolol) [33,34]. Two studies compared FC latanoprost/timolol versus latanoprost and timolol administered concomitantly [35,36].

Four studies compared travoprost and latanoprost [12,13,23,37]. Two studies compared travoprost and timolol [23,38]. Two studies compared travoprost or FC-containing travoprost versus latanoprost (or FC-containing latanoprost) [39,40] and three studies compared travoprost or FC-containing travoprost versus timolol (or FC-containing timolol) [39-41]. Finally, 16 publications contained other comparisons of treatment types that were not duplicated with any other included study. [42-57]

### Characteristics of trials

Table 2 shows the characteristics of the trials as well as the quality of reporting. The average sample size was 311 (SD 33), although on average, only 86% of patients were included in the final analyses. Seventeen out of the included 47 trials (36%, 95% CI: 24–51) were crossover designs.

**Table 2:** Methodological quality and reporting

**Characteristics **	**Number of studies with characteristics**	**% of studies with characteristics**	**95% Confidence Interval**
**Crossover **studies	17	36	24, 51

**Parallel treatment **studies	30	64	49, 76

**Title **and **Abstract **specifying that trial is a noninferiority or equivalence trial	6	13	6, 25

Articles whose **background or introduction section **contains a rationale for using a noninferiority or equivalence trial design	5	11	5, 23

Articles with a clear **objective or hypothesis **pertaining to noninferiority or equivalence	13	28	17, 42

**Sample size determination**: Studies that appropriately described how the sample size was determined*	34	72	58, 83

**Randomization**: Studies stating the methods used in the generation of the random allocation sequence	22	47	33, 61

**Masking:**			
Two or more groups	35	75	60, 85
Patients only	4	9	3, 20
Open label studies (i.e. studies that were not masked)	8	17	9, 30

**Allocation concealment**: Studies reporting this characteristic	10	21	12, 35

**Statistical methods and numbers analyzed:**			
Studies that employed the **combined test of noninferiority and superiority**	8	17	9, 30
Studies presenting results of **only ITT population ***with no mention of PP population*	17	36	24, 51
Studies presenting results of **only PP population ***with no mention of ITT population*	13	28	17, 42
Studies presenting **only results of ITT population ***but mentioned that PP produced similar results*	12	26	15, 39
Studies presenting **only results of PP population ***but mentioned that ITT produced similar results*	2	4	1, 14
Studies reporting and presenting **results of both ITT and PP populations**	3	6	2, 17

**Results:**			
Studies presenting a **figure **showing the *range of the confidence interval relative to the pre-specified noninferiority/equivalence margin*	1	2	0.5, 11

* = appropriate sample size determination stating **all **of the following; a predetermined noninferiority or equivalence margin, standard deviation used, significance level [α], and the power used

Of the 47 trials, 36 were noninferiority trials (77%, 95% CI: 63–86) and 11 (23%, 95% CI: 14–37) were designed for equivalence. In the final interpretations of the studies, 16/47 (34%, 95% CI: 22–48) trials claimed noninferiority, 14/47 (30%, 95% CI: 19–44) claimed equivalence and 17/47 (36%, 95% CI: 24–51) claimed superiority. For the trials claiming noninferiority, 33/36 (92%, 95% CI: 78–92) were accurate within their noninferiority margin. For those claiming equivalency, 10/11 (90%, 95% CI: 62–98) were accurate within their *a priori *margins. Seventeen percent of trials (8/47, 95% CI: 9–30) employed a combined test of noninferiority and superiority.

Sequence generation was reported in 22/47 trials (47%, 95% CI: 33–61). Allocation concealment was reported in only 10/47 (21%, 95% CI: 12–35) of the trials. Thirty-five studies (74%, 95% CI: 60–85) employed masking of at least two groups, 4/47 (9%, 95% CI: 3–20) masked only patients and 8/47 (17%, 95% CI: 9–30) were open label studies.

Only 6 of the 47 (13%, 95% CI: 6–25) studies specified that the trial was a noninferiority or equivalence trial in the title or abstract. The background or introduction section of 5/47 (11%, 95% CI: 5–23) articles contained a rationale for using a noninferiority or equivalence trial design. Thirteen articles (28%, 95% CI: 17–42) had a clear objective or hypothesis pertaining to noninferiority or equivalence. Thirty-four trials (72%, 95% CI: 58–83) properly described the method applied in the sample size determination and set appropriate boundaries on noninferiority or equivalence. The pre-stated noninferiority/equivalence in all the trials lied between the ranges of -1 to 2.5. Primary outcome measure was the mean intraocular pressure which was mostly measured as a mean difference or as a mean reduction from baseline.

Only 3/47 (6%, 95% CI: 2–17) studies reported and presented results of both ITT and PP populations. Two studies (4%, 95% CI: 1–14) presented only PP results but mentioned that ITT population had similar results. Twelve studies (26%, 95% CI: 15–39) presented only ITT results but mentioned that PP population had similar results. Thirteen trials (28%, 95% CI: 17–42) presented only PP results with no mention of ITT population results while 17/47 (36%, 95% CI: 24–51) studies presented only ITT results with no mention of PP population results.

From the 47 trials reported in this study, only one (2%, 95% CI: 0.5–11) diagrammatically presented its results by use of a figure showing the confidence intervals and pre-specified margins of equivalence or noninferiority. Handling of losses to follow-up was not addressed in 33 of the 47 trials (70%, 95% CI: 56–81). Loss to follow-up was mentioned but unaddressed in 10 trials (21%, 95% CI: 12–35), while 3/47 (6%, 95% CI: 2–17) were addressed in the statistics and 1/47 (2%, 95% CI: 0–11) had a zero loss to follow-up.

### Measurement of hyperemia

We found large heterogeneity of measuring hyperemia. Two of the 47 (4%, 95% CI: 1–14) included studies used a severity scale based on non-serious, mild, moderate, and serious. Six studies (13%, 95% CI: 6–25) used a severity scale based on none, mild, moderate, and serious. Five (11%, 95% CI: 5–23) used a scale that recorded only mild, moderate, and severe. One (2%, 95% CI: 0.5–11) used a scale that included only non-serious and serious. One (2%, 95% CI: 0.5–11) used a four-point scale from 1–4 stating minimum and maximum as the scale endpoints. Six (13%, 95% CI:6–25) studies used a 5 point scale with 0 = none, 0.5 = trace, 1 = mild, 2 = moderate, and 3 severe. A further 26/47 (55%, 95% CI: 41–69) did not report their method of measuring hyperemia, of which 5/26 (19%, 95% CI: 9–38) did not report on hyperemia outcomes.

## Discussion

To our knowledge, this is the first study that has attempted to look into the methodological quality and reporting of any ophthalmology RCTs designed to show noninferiority or equivalence for glaucoma treatments. These findings reinforce the gaps that exist in the methodological quality and reporting of these trials in general. We additionally found that within ophthalmology trials, a substantial heterogeneity exists in terms of measuring clinical outcomes, such as hyperemia. It is clear that further efforts at improving the reporting and analyses of clinical trials in this field are needed. Recent work by Hopewell and colleagues [58] highlighted the need for journals to endorse the CONSORT Statement and its extensions, and also make its application a condition for authors wishing to publish in the journal. Journals in the ophthalmology field should consider such an endorsement as well as promoting a strict adherence to the extended CONSORT statement in order to potentially improve manuscript reporting.

There are several strengths and limitations to consider in interpreting our study. Strengths include our extensive searching of the literature to identify a representative sample of a field that commonly utilizes noninferiority and equivalence trials. Our data abstraction methods aimed to reduce investigator driven bias with duplicate data abstraction. Limitations to consider include that we solely relied upon the reporting of specific items rather than inquiring about any missing items through contact with each individual trial investigator. As outlined in the methods, study authors were only contacted when we were unclear whether the trial itself included a non-inferiority or equivalence study design. It is possible that some of the specific methodological items were conducted but not reported. Indeed, this issue has been highlighted by investigators [59]. However, our study aimed to determine reporting quality in order to make specific recommendations on transparent reporting. We did not determine changes pre- versus post-publication of the extended CONSORT statement in 2006. Previous research has found that the quality of reports improved after publication of the original 1996 CONSORT publication [60]. Per-protocol analyses have traditionally been preferred over ITT approaches despite current consensus of displaying both [1,4,7]. In our study we were unable to determine if one is superior to the other as we found too few examples where both outcomes are reported.

The findings of our study are consistent with other studies investigating reporting quality in general medicine journals and specialist journals [61]. General medicine journals tend to report important methodological recommendations more frequently than specialist journals. This may be due to the encouragement of journals to utilize the CONSORT recommendations through editorials and letters [62]. Providing editorials or guidelines in the instructions to authors is a potential option for improving the reporting of these RCTs. An option that may be considered is a checklist submitted with clinical trials indicating where in the manuscript specific details are reported, as required by many current journals.

An important methodological issue that has been raised by this review is the use of a combined test of noninferiority and superiority. The purpose of conducting noninferiority and equivalence trials is not to evaluate superiority, as that would require far more power than would be expected in this type of trial and would maintain the need for ITT analysis [63]. In our study, 8 trials employed the combined test for noninferiority and superiority. This allows a trial to claim its original intent of noninferiority if superiority is not identified, but can claim superiority if the arbitrary threshold, upper-limit of noninferiority, is surpassed [64].

Given the heated market of the prostaglandin field, we found that several (n = 17) noninferiority and equivalence trials claimed superiority of the test intervention over competitors products, regardless of overall findings and the study intent. For example, in a randomized trial evaluating travoprost to latanoprost on 408 patients' daily intraocular pressure at 9 am, 11 am and 4 pm, the authors found noninferiority, but report on statistical superiority at the 9 am measurement [40]. Given the lack of ITT analysis, the multiple testing and initial intention of a noninferiority analysis, such findings provide misleading evaluations of superiority. Indeed, this phenomenon exists across a range of medical fields [65-70].

Pre-specifying the noninferiority or equivalence margin is necessary to provide strong inferences about the suitability of one treatment compared to another. If the margin employed is too wide, risk for type 1 error exists. Similarly, although unlikely, if one were to establish the margin too narrow, a type 2 error is possible. Margins should be established based on clinical justification and not statistical rule [71].

## Conclusion

In conclusion, based on the analysis of the study reports that we have examined, our findings demonstrate deficiencies in the design, planning, and reporting of noninferiority and equivalence trials in the ophthalmology literature. Many studies of clinical noninferiority and equivalence do not set boundaries for equivalence. Claims of "superiority" and "similarity" are often displayed despite the use of improper analysis. These methodologic deficiencies appear to lead to false claims, unrepeatable experiments, and possible costs or harms to patients.

## Competing interests

Over the past 5 years, Edward Mills has consulted to Pfizer Ltd. Edward Mills is the recipient of an industry partnered New Investigator award from The Canadian Institutes of Health Research to conduct methodological research. Chia-Wen Lee has been an employee of Pfizer Ltd. No other authors have any competing interests. We can think of no way that the declared competing interests can benefit from this manuscript.

## Authors' contributions

OE, BR, CWL, and EM conceptualized the study. OE, BR, CWL, and EM acquired the data. OE, BR, CWL, and EM analyzed the data. OE, BR, CWL, and EM wrote the manuscript. OE, BR, CWL, and EM approved the final manuscript.

## Supplementary Material

Additional file 1 Example search strategy, MedLine. Search Strategy for MEDLINE via PubMed Search. Click here for file
