# Supplementary material for: Reporting of noninferiority and equivalence randomized trials for major prostaglandins: A systematic survey of the ophthalmology literature
Source: Trials. 2008 Dec 3;9:69. doi: 10.1186/1745-6215-9-69 (PMC2621118; doi:10.1186/1745-6215-9-69)
Supplement: Additional file 1 — Example search strategy, MedLine. Search Strategy for MEDLINE via PubMed Search. [file 1745-6215-9-69-S1.doc]

***Search Strategy for MEDLINE via PubMed Search***

Using independent and duplicate searchers (OE, EM), we searched MEDLINE via PubMed from its inception to March 2008. We identified the exact MeSH terms for the following expressions: open angle glaucoma, ocular hypertension and prostaglandin* (latanoprost, bimatoprost, travoprost aand unoprostone). We conducted 3 main searches. First we collated all articles with the MeSH *key* terms “glaucoma, open angle OR ocular hypertension”. We then extracted articles containing the MeSH terms “prostaglandin* OR latanoprost OR travoprost OR bimatoprost OR unoprostone” We then pooled these two searches to produce a third list, representing all articles that had as its key terms, “POAG or OH or prostaglandin* OR latanoprost OR travoprost OR bimatoprost OR unoprostone.”
